# Higher-Order Interactions Dampen Pairwise Competition in the Zebrafish Gut Microbiome

**DOI:** 10.1128/mBio.01667-20

**Published:** 2020-10-13

**Authors:** Deepika Sundarraman, Edouard A. Hay, Dylan M. Martins, Drew S. Shields, Noah L. Pettinari, Raghuveer Parthasarathy

**Affiliations:** a Department of Physics, University of Oregon, Eugene, Oregon, USA; b Institute of Molecular Biology, University of Oregon, Eugene, Oregon, USA; c Materials Science Institute, University of Oregon, Eugene, Oregon, USA; Carnegie Institution for Science; University of Hawaii at Manoa

**Keywords:** gut microbiome, microbial interactions, zebrafish, gnotobiotic

## Abstract

The microbial communities resident in animal intestines are composed of multiple species that together play important roles in host development, health, and disease. Due to the complexity of these communities and the difficulty of characterizing them *in situ*, the determinants of microbial composition remain largely unknown. Further, it is unclear for many multispecies consortia whether their species-level makeup can be predicted based on an understanding of pairwise species interactions or whether higher-order interactions are needed to explain emergent compositions. To address this, we examine commensal intestinal microbes in larval zebrafish, initially raised germfree, to allow the introduction of controlled combinations of bacterial species. Using a dissection and plating assay, we demonstrate the construction of communities of one to five bacterial species and show that the outcomes from the two-species competitions fail to predict species abundances in more complex communities. With multiple species present, interbacterial interactions become weaker, suggesting that higher-order interactions in the vertebrate gut stabilize complex communities.

## Abstract (precis)

Understanding the rules governing the composition of the diverse microbial communities that reside in the vertebrate gut environment will enhance our ability to manipulate such communities for therapeutic ends. Synthetic microbial communities, assembled from specific combinations of microbial species in germfree animals, allow investigation of the fundamental question of whether multispecies community composition can be predicted solely based on the combined effects of interactions between pairs of species. If so, such predictability would enable the construction of communities with desired species from the bottom up. If not, the apparent higher-order interactions imply that emergent community-level characteristics are crucial. Our findings using up to five coexisting native bacterial species in larval zebrafish, a model vertebrate, provide experimental evidence for higher-order interactions and, moreover, show that these interactions promote the coexistence of microbial species in the gut.

## INTRODUCTION

Intestinal microbes exist in complex and heterogeneous communities of interacting, taxonomically diverse species. The composition of these communities varies across individuals and is crucial to the health of the host, having been shown in humans and other animals to be correlated with dietary fat uptake (1, 2), organ development (3, 4), immune regulation (5–10), and a wide range of diseases (11–20).

Despite the importance of intestinal communities, the determinants of their composition remain largely unknown. A growing number of studies map the effects of external perturbations, such as antibiotic drugs (21, 22) and dietary fiber (23) and fat (24, 25), on the relative abundance of gut microbial species. Intrinsic intermicrobial interactions, however, are especially challenging to measure and are important not only for shaping community composition in the absence of perturbations but also for propagating species-specific perturbations to the rest of the intestinal ecosystem.

The considerable majority of studies of the gut microbiota have been performed on naturally assembled microbiomes by sequencing DNA extracted from fecal samples, an approach that provides information about the microbial species and genes present in the gut but that imposes several limitations on the inference of interspecies interactions. The high diversity of natural intestinal communities and, therefore, the low abundance of any given species among the multitude of its fellow residents implies that stochastic fluctuations in each species’ abundance will be large, easily masking true biological interactions. The accuracy of inference is considerably worse if only relative, rather than absolute, abundance data are available (26–29), as is typically the case in sequencing-based studies. Finally, we note that fecal sampling assesses only the microbes that have exited the host, which may not be representative of the intestinal community (30).

**FIG 1:**
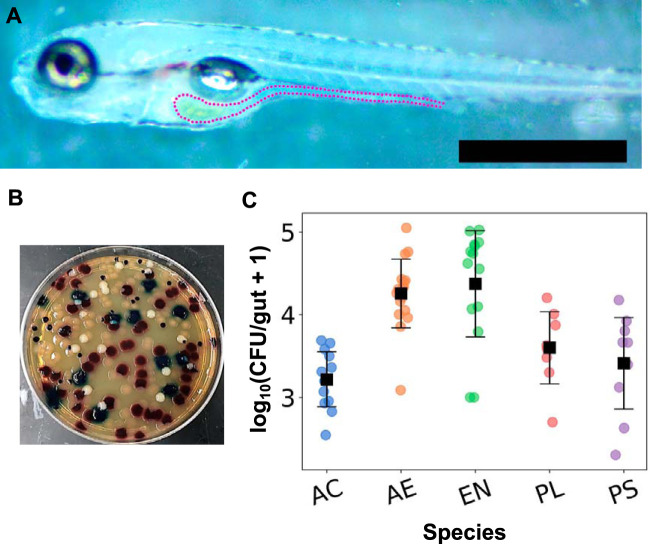
Five chosen commensal species are robust colonizers of the larval zebrafish intestine. (A) A 7-dpf larval zebrafish, with a dotted line outlining the intestine. Scale bar, 500 μm. (B) Chromogenic agar plate showing colonies of all five candidate species (A. calcoaceticus, milky opaque; *Aeromonas* sp. strain ZOR0001, reddish purple; *Enterobacter* sp. strain ZOR0014, blue; *Plesiomonas* sp. strain ZOR0011, dark purple; P. mendocina, colorless translucent). (C) The abundance per zebrafish gut of each of the five bacterial species when colonized in monoassociation with the host, assessed as number of CFU from plated gut contents. Each circular data point is a CFU value from an individual fish (*N* = 13, 17, 15, 8, and 10, from left to right), with the mean and standard deviation indicated by the square markers and error bars. AC, Acinetobacter calcoaceticus; AE, *Aeromonas* sp. strain ZOR0001; EN, *Enterobacter* sp. strain ZOR0014; PL, *Plesiomonas* sp. strain ZOR0011; PS, Pseudomonas mendocina.

An alternative approach to using DNA sequencing and naturally assembled host-microbiota systems is to build such systems from the bottom up using model organisms. This is accomplished by using techniques for generating initially germfree animals, and well-defined sets of small numbers of microbial species, and then measuring the populations of these species resident in the intestine. Recent work along these lines has been performed using the nematode Caenorhabditis elegans (31) and the fruit fly Drosophila melanogaster (32, 33). However, as described further below, these studies imply different principles are at play in the different systems. Moreover, it is unclear whether conclusions from either model platform translate to a vertebrate gut, which has both greater anatomical complexity and more specific microbial selection (34). To address this, we measure bacterial interactions in larval zebrafish (Fig. 1A), a model vertebrate organism amenable to gnotobiotic techniques (35–38), which enabled, in earlier works that investigated pairs of bacterial species, the discovery of specific interbacterial competition mechanisms related to intestinal transport (39, 40). The experiments described here involve several hundred fish, each with 1 to 5 resident bacterial species, enabling robust inference of interspecies interactions.

The ability to quantify species abundance and to manipulate it by controlled addition or subtraction of species is commonplace in macroscopic ecological investigations. Its implementation here enables connections between intestinal microbiome research and a large literature on ecosystem dynamics. An issue whose importance has been realized for decades is the extent to which interspecies interactions are pairwise additive or whether higher-order (often called indirect) interactions are necessary to explain community structure (41, 42). The term “higher-order interactions” has been defined in various ways in the ecological literature (42, 43), in some cases referring specifically to nonadditive changes in a species’ growth rate given the presence of additional species, or to changes in the nature of the interaction between two species induced by additional species. In other cases it refers more generally to any interaction that cannot be captured by a pairwise model. We adopt the latter, commonly used definition, which is agnostic to underlying mechanisms (44). In our analyses below, we consider various pairwise models and assess their ability to describe data from multispecies communities; mismatch is indicative of the existence of higher-order interactions. Pairwise additivity, if dominant, simplifies the prediction of ecosystem composition, which would be desirable for therapeutic applications of microbiome engineering. Higher-order interactions may stabilize multispecies communities according to several recent theoretical models described further in Discussion (45–48), implying that quantifying and controlling indirect effects is necessary for reshaping gut microbiomes.

Whether host associated or not, microbial communities have shown a variety of interaction types. A classic study involving cultured protozoan species found good agreement between the dynamics of four-species consortia and predictions derived from measurements of pairs of species (49). Similarly, Friedman and colleagues showed that the outcomes of competitions among three-species communities of soil-derived bacteria could be predicted simply from the outcomes of pairwise combinations (50). In contrast, experiments based on the cheese rind microbiome found significant differences in the genes required for a nonnative Escherichia coli species to persist in a multispecies bacterial community compared to predictions from pairwise coexistence with community members (51). A closed ecosystem consisting of one species each of algae, bacteria, and ciliate exhibited a strong nonpairwise interaction, in which the bacteria is abundant in the presence of each of the algae or ciliate alone but is subject to strong predation in the three-species system (52).

Within animals, the interaction types observed in the few studies to date that make use of controlled microbial communities in gnotobiotic hosts are also disparate. Competitive outcomes of three-species communities from subsets of 11 different bacterial species in the gut of the nematode C. elegans could be predicted from the outcomes of two-species experiments, with indirect effects found to be weak (31). In contrast, work using well-defined bacterial assemblies of up to five species in the fruit fly D. melanogaster found strong higher-order interactions governing microbe-dependent effects on host traits, such as life span (32).

To our knowledge, there have been no quantitative assessments of interbacterial interactions using controlled combinations of microbial species in a vertebrate host, leaving open the question of whether higher-order interactions are strong or whether pairwise characterizations suffice to predict intestinal community structure. Therefore, we examined larval zebrafish, inoculating initially germfree animals with specific subsets of five different species of zebrafish-derived bacteria and assessing their subsequent absolute abundances. Although the number of species is considerably smaller than the hundreds that may be present in a normal zebrafish intestine, it is large enough to sample a range of higher-order interactions yet small enough that the number of permutations of species is tractable.

As detailed below, we find strong pairwise interactions between certain bacterial species. However, we find weaker interactions and a greater than expected level of coexistence in fish colonized by four or five bacterial species. This suggests that measurements of pairwise intermicrobial interactions are insufficient to predict the composition of multispecies gut communities and that higher-order interactions dampen strong competition and facilitate diversity in a vertebrate intestine.

## RESULTS

Zebrafish (Fig. 1A) were derived to be germfree and then were inoculated at 5 days postfertilization (dpf) with the desired combination of microbial species by addition of bacteria to the flasks housing the fish. Approximately 48 h later, fish were euthanized and their intestines removed by dissection. Intestines and their contents were homogenized, diluted, and plated onto chromogenic agar (see Materials and Methods). Secreted enzymes from each of the five candidate bacterial species generate particular colors due to substrates in the chromogenic medium, allowing the quantification of the number of CFU and, therefore, absolute intestinal abundance (Fig. 1B). All abundance data are provided in Data Set S1 in the supplemental material.

10.1128/mBio.01667-20.1 DATA SET S1 Absolute abundance data for all experiments. Spreadsheet containing absolute counts of bacterial cells observed for each of the species for each fish inoculated in 1-, 2-, 4-, and 5-species *in vivo* and 1- and 2-species *in vitro* experiments. Data from each experiment are in a separate sheet with the experiment’s name, e.g., “2-species (*in vivo*).” Within each sheet, the titles of the columns indicate the species combination inoculated for a particular experiment. Different species combinations are separated by empty columns. Download Data Set S1, XLSX file, 0.03 MB. Copyright © 2020 Sundarraman et al. 2020 Sundarraman et al. This content is distributed under the terms of the Creative Commons Attribution 4.0 International license.

The five species examined were selected as diverse representatives of genera commonly found in the zebrafish intestine. Full names and species identifiers are given in Materials and Methods. As expected given their association with the zebrafish gut microbiome, each species in monoassociation, i.e., as the sole species inoculated in germfree fish, colonizes robustly to an abundance of 10^3^ to 10^4^ CFU/gut, corresponding to an *in vivo* density of approximately 10^9^ to 10^10^ bacteria/ml (Fig. 1C).

### Pairwise interactions in diassociations.

**FIG 2:**
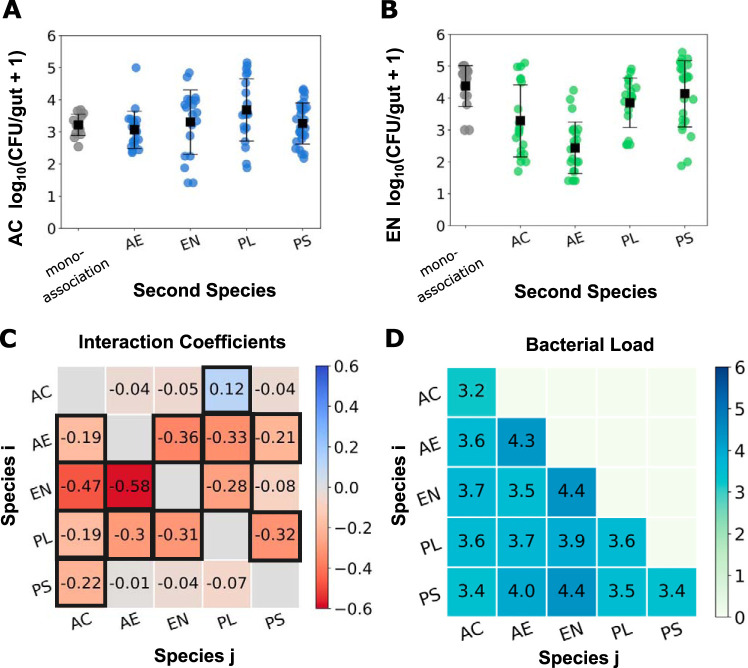
Strong negative pairwise interactions dominate diassociation experiments. Abundances per zebrafish gut of A. calcoaceticus (A) and *Enterobacter* sp. strain ZOR0014 (B) in monoassociation (gray) and in diassociation with each of the other bacterial species (blue/green). Each circular data point is a CFU value from an individual fish (*N* = 13, 21, 19, 20, and 27 [A] and *N* = 15, 19, 22, 18, and 23 [B], from left to right), with the means and standard deviations indicated by the square markers and error bars. (C) Matrix of pairwise interaction coefficients, $C_{\mathrm{ij}}^{\mathrm{II}}$, characterizing the effect of species *j* on the abundance of species *i*. Coefficients that differ from zero by more than three standard deviations (provided in Fig. S2A) are outlined in black. (D) The average bacterial load per zebrafish in each of the diassociation combinations, expressed as log_10_ total CFU. The standard deviations are between 0.3 and 1.1 and are displayed in Fig. S1. Values on the diagonal are the monoassociation load for each of the five species. AC, Acinetobacter calcoaceticus; AE, *Aeromonas* sp. strain ZOR0001; EN, *Enterobacter* sp. strain ZOR0014; PL, *Plesiomonas* sp. strain ZOR0011; PS, Pseudomonas mendocina.

We first examined all 10 possible coinoculations of two species, which enables the assessment of pairwise interactions in the absence of higher-order effects. Intestinal CFU data show a wide range of outcomes for different species pairs. As exemplars, the number of CFU per gut for each of two species, Acinetobacter calcoaceticus and *Enterobacter* sp. strain ZOR0014, in the presence of each of the other four is displayed in Fig. 2A and B, respectively. The abundance of A. calcoaceticus is similar in the presence of any second species to its value in monoassociation. In contrast, the mean *Enterobacter* sp. strain ZOR0014 abundance is similar to its monoassociation value if coinoculated with *Plesiomonas* sp. strain ZOR0011 or Pseudomonas mendocina, about 10 times lower if coinoculated with A. calcoaceticus, and over 2 orders of magnitude lower if coinoculated with *Aeromonas* sp. strain ZOR0001, implying in the latter cases strong negative interactions.

10.1128/mBio.01667-20.4 FIG S1 Total bacterial load for different diassociation experiments. The total bacterial load for different diassociation experiments, expressed as the mean and standard deviation of log_10_(total CFU). The values on the diagonal are the mean load from monoassociation experiments. Download FIG S1, PDF file, 0.2 MB. Copyright © 2020 Sundarraman et al. 2020 Sundarraman et al. This content is distributed under the terms of the Creative Commons Attribution 4.0 International license.

10.1128/mBio.01667-20.5FIG S2Pairwise interaction coefficients for two-species and five-species experiments using the log-transformed model. The mean pairwise interaction coefficients, $C_{\mathrm{ij}}^{\mathrm{II}}$ (A) and $C_{\mathrm{ij}}^{V}$(B), showing the effect of species *j* on species *i*, calculated using the log-transformed abundance model. The standard deviations were calculated using a subsampling approach as described for Text S1. Download FIG S2, PDF file, 0.6 MB.Copyright © 2020 Sundarraman et al.2020Sundarraman et al.This content is distributed under the terms of the Creative Commons Attribution 4.0 International license.

Parameterizing the strength of interactions between species is necessarily model dependent, contingent on the functional form of the relationship between one species’ abundance and that of the other. We show that the conclusions we reach regarding interaction strengths, especially their shifts when multiple species are present, are qualitatively similar for a wide range of models and, therefore, robust. We first consider a phenomenological interaction coefficient, $C_{\mathrm{ij}}^{\mathrm{II}}$, that is linear in log abundance, characterizing the effect of species *j* on species *i* as

(1) $${\text{log}}_{10}\;P_{i}^{\mathrm{II}}=〈{\text{log}}_{10}\;P_{i}^{I}〉+C_{\mathrm{ij}}^{\mathrm{II}}{\text{log}}_{10}\;P_{j}^{\mathrm{II}}$$

where *P_i_* denotes the abundance of species *i* and the superscript I or II denotes a mono- or diassociation experiment. This form is motivated by the distribution of gut bacterial abundances being roughly lognormal, with species addition capable of inducing orders-of-magnitude changes (Fig. 2A and B). This $C_{\mathrm{ij}}^{\mathrm{II}}$ value can be derived as the interaction parameter in a competitive Lotka-Volterra model modified to act on log abundances (see Text S1). Qualitatively, a positive *C_ij_* implies that the abundance of species *i* increases in the presence of *j*. Similarly, a negative *C_ij_* indicates that the abundance of species *i* declines in the presence of species *j*. Subsampling from the measured sets of bacterial abundances gives the mean and standard deviation of the estimated interaction parameters (Text S1).

10.1128/mBio.01667-20.10 TEXT S1 Math supplement. File containing a detailed description of the different interaction models and the procedures followed for carrying out the analysis of the experimental data. Download Text S1, PDF file, 0.1 MB. Copyright © 2020 Sundarraman et al. 2020 Sundarraman et al. This content is distributed under the terms of the Creative Commons Attribution 4.0 International license.

We plot in Fig. 2C the $C_{\mathrm{ij}}^{\mathrm{II}}$ defined by Equation 1, calculated from all diassociation data of all species pairs (*N* = 190 fish in total). For determining $C_{\mathrm{ij}}^{\mathrm{II}}$, we only use data from fish in which both species were detected so that abundance changes of one species can definitively be ascribed to the presence of the other within the gut. Uncertainties in $C_{\mathrm{ij}}^{\mathrm{II}}$ are estimated from bootstrap subsampling (Text S1). The interactions are predominantly negative. Thirteen out of 20 coefficients differ from zero by over three standard deviations, indicating both a large magnitude and a less than 0.001 probability that the interaction strength is zero or of the opposite sign. The total bacterial load, i.e., the sum of the bacterial abundances, is similar for all the diassociations, suggesting that the interaction effects do not stem from changes in intestinal capacity (Fig. 2D).

**FIG 3:**
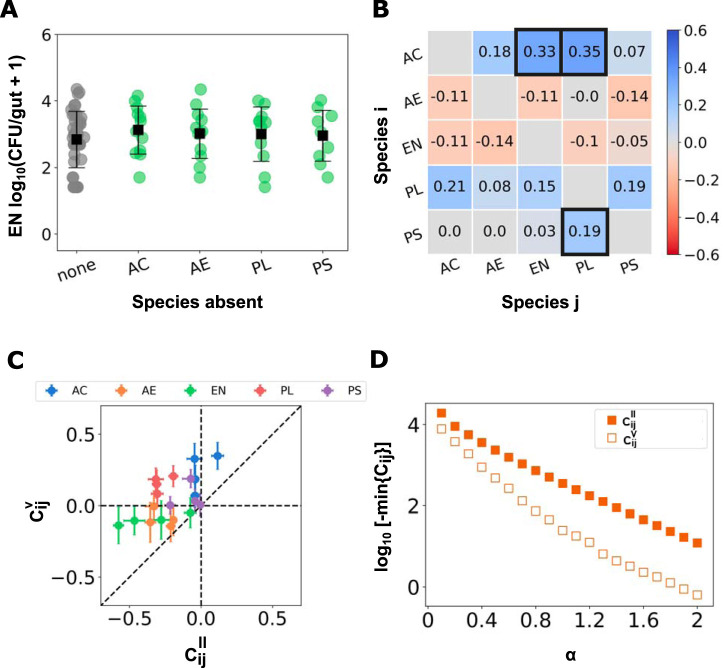
Weak pairwise interactions in five-species experiments. (A) Abundance per zebrafish gut of one of the bacterial species, *Enterobacter* sp. strain ZOR0014, when all five species are coinoculated (gray) and in each four species coinoculation experiments (green), with the omitted species indicated on the axis. Each circular data point is a CFU value from an individual fish (*N* = 40, 12, 12, 11, and 9, from left to right), with the mean and standard deviation indicated by the square marker and error bars. (B) Matrix of pairwise interaction coefficients, $C_{\mathrm{ij}}^{V}$, when 5 bacterial species are present. The coefficients outlined in black differ from zero by over three standard deviations (see Fig. S2B). (C) The pairwise interaction coefficients inferred from 4- to 5-species experiments versus those from 1- to 2-species experiments. The colors label species *i* for each interaction pair. (D) The minimum interaction coefficient calculated from a power law interaction model for different values of the exponent *α* for the 1- to 2-species (square filled markers) and the 4- to 5-species (square markers) experiments. AC, Acinetobacter calcoaceticus; AE, *Aeromonas* sp. strain ZOR0001; EN, *Enterobacter* sp. strain ZOR0014; PL, *Plesiomonas* sp. strain ZOR0011; PS, Pseudomonas mendocina.

Although the physical and chemical environment of the zebrafish gut is likely very dissimilar to test tubes of standard growth media, we examined abundances of each of the pairs of species in *in vitro* competition experiments, growing overnight cultures in lysogeny broth (LB) medium and plating for CFU (see Materials and Methods). Assessing $C_{\mathrm{ij}}^{\mathrm{II}}$ as described above, we find, as expected, that interaction coefficients calculated from the *in vitro* experiments are markedly different from those measured *in vivo* (Fig. 3B and Fig. S3).

10.1128/mBio.01667-20.6FIG S3Pairwise interaction coefficients from *in vitro* two-species experiments calculated using the log-transformed abundance model. The matrix of interaction coefficients shows the mean and standard deviation of $C_{\mathrm{ij}}^{\mathrm{II}}$ from *in vitro* competition experiments. Download FIG S3, PDF file, 0.3 MB.Copyright © 2020 Sundarraman et al.2020Sundarraman et al.This content is distributed under the terms of the Creative Commons Attribution 4.0 International license.

Our characterizations of interactions within the zebrafish gut are not qualitatively altered by using a more general power law model to compute $C_{\mathrm{ij}}^{\mathrm{II}}$ from absolute abundance data (see “Interactions under more general models,” below) following the presentation of measurements of interactions between more than two species.

### Pairwise interactions in multispecies communities.

To assess whether the strong competitive interactions we found in two-species experiments are conserved in multispecies communities, we quantified pairwise interactions in experiments inoculating fish with four or five bacterial species. To assess $C_{\mathrm{ij}}^{V}$, we adopted a method similar to the leave-one-out approach often used in macroscopic ecological studies, dating at least to classic experiments in which single species were removed from tide pools and the abundances of the remaining species were measured to evaluate interspecies interactions (53). Here, we performed coinoculation experiments, leaving out one of the five species of bacteria, and compared intestinal abundances for these four-species communities to those measured in five-species coinoculation experiments.

In approximately *N* = 10 fish each, we performed all five different coinoculations of four bacterial species. The difference in the abundance of species *i* in fish inoculated with all five species compared to fish inoculated with four, missing species *j*, gives a measure of the impact of species *j* on species *i* in the multispecies environment. As an example, *Enterobacter* sp. strain ZOR0014 abundance in inoculations lacking A. calcoaceticus, *Aeromonas* sp. strain ZOR0001, *Plesiomonas* sp. strain ZOR0011, and P. mendocina, and its abundance in five-species inoculations, are shown in Fig. 3A. In contrast to the diassociation experiments (Fig. 2B), we see that *Enterobacter* sp. strain ZOR0014 does not show large abundance differences, in either its mean or its distribution, as a result of any fifth species being present. Independent of any model, this suggests that nonpairwise, i.e., higher-order, interactions are present in the multispecies community.

Again, a variety of options are possible for quantifying interaction coefficients in the multispecies system. We first consider interaction coefficients as modifying mean log abundances, analogous to the pairwise model of Equation 1:

(2) $${\text{log}}_{10}\;P_{i}^{V}={\text{log}}_{10}\;P_{i}^{IV}+C_{ij}^{V}\;{\text{log}}_{10}\;P_{j}^{V}$$

The interaction coefficients, $C_{\mathrm{ij}}^{V}$, that we obtain, displayed in Fig. 3B, are different and in general are considerably weaker than the $C_{\mathrm{ij}}^{\mathrm{II}}$ found in the two-species case (Fig. 2C). There are only three interactions that differ from zero by over three standard deviations. Strikingly, all three of these interactions are positive. This shift toward weaker and more positive interactions between the two-species and multispecies interactions is further illustrated in Fig. 3C, in which the multispecies interaction coefficients, $C_{\mathrm{ij}}^{V}$, are plotted against the 2-species interaction coefficients, $C_{\mathrm{ij}}^{\mathrm{II}}$.

### Interactions under more general models.

As noted, a model that is linearly additive in logarithmic abundances is only one of an infinite number of choices and, moreover, may not adequately capture the complexity of interactions in the gut. Earlier experiments investigating the spatial structure of specific microbial communities in the larval zebrafish intestine have shown that species such as *Aeromonas* sp. strain ZOR0001, *Enterobacter* sp. strain ZOR0014, and P. mendocina form dense three-dimensional aggregates (54). The size and location of aggregates and the locations of cells, conspecific or otherwise, within these aggregates may affect their interactions in ways that could be sublinear, linear, or superlinear in population size. Previous work has also established that gut bacteria also influence intestinal mechanics (40), highlighting one of many possible indirect interaction mechanisms whose functional forms are unknown. Furthermore, other studies have shown that different modes of physical and chemical communication could result in long-range interactions between different species (55–57). To address these possibilities, we evaluated species interactions with a more general power law model, wherein the interaction effects between species could be nonlinear in the abundance of the effector species. Here, the interaction coefficient *C_ij_* depends on a power, *α*, of the abundance of the effector species *j*, which we evaluate in the range of *α* = 0.1 to 2, spanning sublinear and superlinear interactions. Modified versions of Equations 1 and 2 give

(3) $$P_{i}^{\mathrm{II}}=〈P_{i}^{I}〉+C_{ij}^{II}{\left(P_{j}^{V}\right)}^{\alpha }$$

and

(4) $$P_{i}^{V}=〈P_{i}^{IV}〉+C_{ij}^{V}{\left(P_{j}^{V}\right)}^{\alpha }$$

from which we can evaluate $C_{ij}^{II}$ and $C_{ij}^{V}$, respectively. Note that *α* = 1 in Equations 3 and 4, i.e., interactions that are linear in abundance, is simply the steady-state behavior of the competitive Lotka-Volterra model commonly used in population modeling and is shown in Fig. S5. We provide the $C_{\mathrm{ij}}^{\mathrm{II}}$ and $C_{\mathrm{ij}}^{V}$ for several different *α* values in Fig. S4. Throughout, as in the logarithmic model shown above, pairwise interactions in diassociation are, in many cases, strongly negative, while the multispecies interactions are weaker. This is summarized by studying the trends in the most negative $C_{\mathrm{ij}}^{\mathrm{II}}$ and $C_{\mathrm{ij}}^{V}$ for different values of *α*, depicted in Fig. 3D, which shows that for all *α* values, the strongest $C_{\mathrm{ij}}^{V}$ is significantly weaker than the strongest $C_{\mathrm{ij}}^{\mathrm{II}}$, suggesting that our results are robust to choice of model.

10.1128/mBio.01667-20.7 FIG S4 Pairwise interaction coefficients for select *α* values for two- and five-species experiments using the power law model. Interaction coefficients generated from the linear absolute abundance model are compared for the one- to two-species (left) and four- to five-species (right) experiments. The legend at the bottom right shows the species labels for the rows and columns. The data are tabulated in Data Set S2. Download FIG S4, PDF file, 0.9 MB. Copyright © 2020 Sundarraman et al. 2020 Sundarraman et al. This content is distributed under the terms of the Creative Commons Attribution 4.0 International license.

10.1128/mBio.01667-20.2DATA SET S2Pairwise interaction coefficients for two-species ($C_{\mathrm{ij}}^{\mathrm{II}}$) and for five-species ($C_{\mathrm{ij}}^{V}$) experiments calculated from the power law model. The spreadsheet contains the interaction coefficients for the 2-species and 5-species experiments calculated using the power law model for different values of *α*. Each sheet contains the mean and standard deviation of the coefficients ($C_{\mathrm{ij}}^{\mathrm{II}}$ and $C_{\mathrm{ij}}^{V}$) for a single value of *α*. The first column in each sheet depicts the species pair (*i-j*). Download Data Set S2, XLSX file, 0.02 MB.Copyright © 2020 Sundarraman et al.2020Sundarraman et al.This content is distributed under the terms of the Creative Commons Attribution 4.0 International license.

10.1128/mBio.01667-20.8FIG S5Pairwise interaction coefficients for two- and five-species experiments using a linear model. The mean and standard deviation of the interaction coefficients for the two-species ($C_{\mathrm{ij}}^{\mathrm{II}}$) and five-species ($C_{\mathrm{ij}}^{V}$) experiments calculated using a linear model in absolute species abundance. Download FIG S5, PDF file, 0.5 MB.Copyright © 2020 Sundarraman et al.2020Sundarraman et al.This content is distributed under the terms of the Creative Commons Attribution 4.0 International license.

### Five-species coexistence.

**FIG 4:**
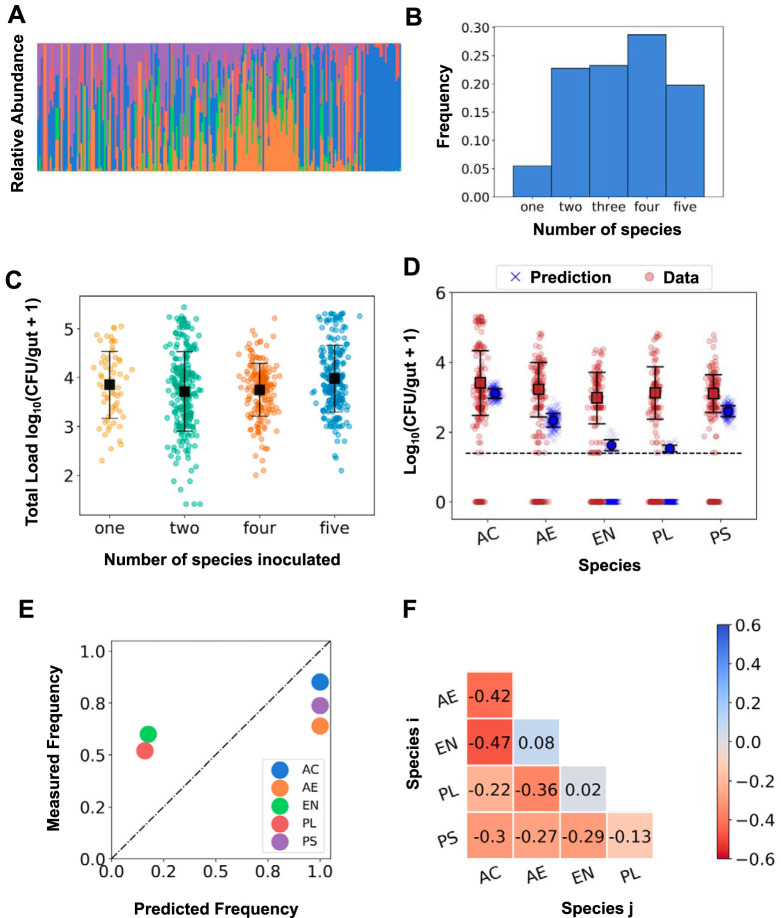
Communities are more diverse and abundant in five-species experiments than would be predicted solely based on two-species pairwise interactions. (A) Stacked bar plot of the relative abundances of the five bacterial species when all five were coinoculated. Each bar is from a single dissected fish. The bars are ordered by total bacterial load. (B) Histogram of the total number of bacterial species present in the gut when all five species were coinoculated. (C) The total bacterial load as a function of the number of inoculated species. Each circular data point is a CFU value from an individual fish (*N* = 63, 232, 187, and 202, from left to right), with the mean and standard deviation indicated by the square marker and error bars. (D) The predicted (blue Xs) and measured (brown circles) abundances of each bacterial species in the zebrafish gut when all five species are coinoculated. Predictions are based on an interaction model that is linear in log abundance using the pairwise $C_{\mathrm{ij}}^{\mathrm{II}}$ coefficients, as described in the text. Solid square markers indicate the mean and standard deviation of the distributions excluding null counts. The dotted line indicates the experimental detection limit of 25 cells. The experimental data are from *N* = 202 fish in total, and the predicted distributions arise from 250 samples of the distribution of interaction coefficients. (E) The observed frequency of occurrence in the gut from the five-species coinoculation experiment versus the predicted frequencies for each of the five species. (F) The Pearson correlation coefficients calculated from the relative abundances of pairs of species when all five species were coinoculated. AC, Acinetobacter calcoaceticus; AE, *Aeromonas* sp. strain ZOR0001; EN, *Enterobacter* sp. strain ZOR0014; PL, *Plesiomonas* sp. strain ZOR0011; PS, Pseudomonas mendocina.

We next consider coinoculation of all five bacterial species. Examination of over 200 fish shows a large variety in abundances, depicted in Fig. 4A as the relative abundance of each species in each larval gut. Multiple species are able to coexist, with the median number of species present being 4 (Fig. 4B). The mean total bacterial load as well as its distribution (Fig. 4C) are similar to the mean and distribution of the mono- and diassociation experiments, as well as four-species coinoculation experiments discussed earlier. We calculated the expected abundance of each bacterial species if the interactions governing the five-species community were simply a linear combination of the pairwise interactions governing diassociations, $C_{\mathrm{ij}}^{\mathrm{II}}$. Any of the additive models we evaluated can be extended to combinations of species. Considering the model focused on above, with interaction coefficients modifying log abundances, the predicted abundance of species *i* in the presence of another species *j* is given by

(5) $${\text{log}}_{10}\;P_{i}^{V}=〈{\text{log}}_{10}\;P_{i}^{I}〉+\underset{j\neq i}{\sum }C_{ij}^{II}\;{\text{log}}_{10}\;P_{j}^{V}$$

where the superscript *V* denotes the five-species coinoculation experiment. A model linear in species abundance (*α* = 1) is also considered in the Text S1 and gives qualitatively similar outputs and conclusions. Sampling from the measured distributions of each of the interaction coefficients and the mean abundance in monoassociation allows the calculation of the distribution of expected $P_{i}^{V}$ values (see Data Set S3).

10.1128/mBio.01667-20.3 DATA SET S3 Predicted abundance distributions for log-transformed abundance model. The file contains the predicted five-species abundances of species using the linear additive model described in the text for the log-transformed abundance model (Sheet 1) and the absolute abundance model (Sheet 2). Each row within each sheet represents the predicted abundance of species A. calcoaceticus, *Aeromonas* sp. strain ZOR0001, *Enterobacter* sp. strain ZOR0014, *Plesiomonas* sp. strain ZOR0011, and P. mendocina in one fish. Download Data Set S3, XLSX file, 0.04 MB. Copyright © 2020 Sundarraman et al. 2020 Sundarraman et al. This content is distributed under the terms of the Creative Commons Attribution 4.0 International license.

We plot the measured and predicted distributions of intestinal abundances of each of the five species for the five-species coinoculation experiment in Fig. 4D. The measured distributions of each of the species are very similar. In contrast, the distributions of the predicted abundances vary significantly by species. For two of the species, A. calcoaceticus and P. mendocina, the means of the observed and predicted distributions are similar. For the other three species, in contrast, the observed and predicted populations are in strong disagreement, with the pairwise prediction being at least an order of magnitude lower than the observed abundances. For *Enterobacter* sp. strain ZOR0014 and *Plesiomonas* sp. strain ZOR0011 in particular, we would expect extinction in a large fraction of fish due to strong negative pairwise interactions; in actuality, both species are common and abundant.

Similarly, we can extract from the model the predicted frequency of occurrence of each of the species, regardless of abundance, i.e., the fraction of fish with a nonzero population of that species. We find that the predicted frequency is much lower than the experimentally observed frequency for *Plesiomonas* sp. strain ZOR0011 and *Enterobacter* sp. strain ZOR0014 (Fig. 4E).

By measuring absolute abundances of bacterial populations in the gut, we provide direct assessments of interspecies interactions. More common sequencing-based methods, applied, for example, to the human gut microbiome, typically provide relative measures of species abundance, i.e., each taxonomic unit’s fraction of the total load. Correlations among relative abundances are often used as measures of interaction strengths (58, 59). Calculating the Pearson correlation coefficients of the relative abundances of each pair of species in fish inoculated with all five bacterial species, we find a strikingly different interaction matrix (Fig. 4F) than that inferred from absolute abundance changes (Fig. 3B), with many strong negative values. There are many likely reasons for the difference between Pearson correlations and our more directly measured interaction coefficients. The Pearson *r* necessarily attributes correlations between pairs of species as being indicative of the dynamics of that pair independent of other species, is confounded by overall changes in total bacterial load, and, perhaps most importantly, is necessarily symmetric (*C_ij_* = *C_ji_*). Our *C_ij_* values, inferred from absolute abundance data, are notably asymmetric (Fig. 2C and 3B).

## DISCUSSION

Using a model system comprising five commensal bacterial species in the larval zebrafish intestine, we have characterized aspects of gut microbiome assembly. Controlled combinations of inoculated species and measurements of absolute abundance in the gut, both challenging to perform in other vertebrate systems, reveal clear signatures of interactions among species. We find strong, competitive interactions among certain pairs in fish inoculated with two bacterial species. In contrast, pairwise interactions are weak in intestines colonized by four to five species, and all species are present at equal or greater abundance than would be predicted based on two-species data.

Our quantification of interaction strengths relies on a minimal set of assumptions that serve as a general test of additive models. Interaction strengths are necessarily parameters of particular models. We made use of a model in which the log-transformed population of a species is a linear function of the other species’ log-transformed populations and a more general power law model that spans both sublinear and superlinear dependences on population sizes. There are good reasons to be skeptical of such frameworks. First, intestinal populations may not be well described by equilibrium, steady-state values. Second, these models lack spatial structure information. *In vivo* microscopy of one or two species in the zebrafish gut (39, 40, 60) underscores both of these concerns: populations are very dynamic, with rapid growth and stochastic expulsions; interactions can be mediated by complex intestinal mechanics; and aggregation and localization behaviors are species specific.

Imaging, however, provides justifications for these rough models. Prior microscopy-based studies have shown that growth rates are rapid, with populations reaching carrying capacities within roughly 12 h (39, 60), well below the 48-h assessment time considered here. Because of strong aggregation observed in nearly all bacterial species, most individual bacteria residing in the bulk of clusters will not directly interact with other species, leading to interactions that are sublinear in population size, suggesting a logarithmic or *α* < 1 power law functional form. Furthermore, stochastic dynamics can be mapped onto robust average properties for populations (39, 61). Therefore, it is reasonable to make use of simple models not as rigorous descriptions of the system but as approximations whose parameters characterize effective behaviors. We note that all these issues also affect more commonly used models, such as standard competitive Lotka-Volterra models that are linear in population sizes. These models are often applied to gut microbiome data and used to infer interaction parameters (26, 62, 63) despite a lack of information about their realism. The power law model of interactions provides the strongest indication of the generality of our conclusions. Over a range of interaction forms extending from highly sublinear (*α* = 0.1) to superlinear (*α* = 2.0), strong competitive interactions are dampened when four or five species are present (Fig. 3D), suggestive of higher-order interactions among intestinal bacteria.

The ecological potential for higher-order or indirect interactions, i.e., interactions that cannot be reduced to pairwise additive components but rather result from the activities of three or more species, to be important determinants of community structure has been appreciated for decades (41, 42, 49). Identification of higher-order interactions among constituent species is important for accurate prediction of responses to ecological perturbations such as species invasion or extinction, as well as functions of multispecies communities, as such features will not be adequately forecast by the examination of direct interactions in subsystems (41, 64).

Inferring and quantifying indirect interactions in natural ecosystems is, however, challenging, calling for subtle and model-dependent statistical tests (41, 42, 65). Constructed or manipulated systems enable more straightforward assays in which particular species are introduced or removed amid a backdrop of others. Several such systems involving macroscopic organisms (66–70), as well as microorganisms (32, 52), have uncovered significant indirect interactions. However, some studies of microbial communities have found weak or negligible higher-order interactions (49, 50), including one study examining combinations of species introduced to the C. elegans gut (31). The complexity of interactions in a vertebrate gut has remained unclear, and correlation-based methods for inferring interactions from sequencing-based data have assumed that pairwise interactions suffice (58, 71, 72).

Our measurements using gnotobiotic larval zebrafish, a model vertebrate, show strong pairwise interactions when only two bacterial species are present in the intestine and weak pairwise interactions when four to five species are present, indicating that higher-order interactions are important (Fig. 3). In many cases, the effect is evident from the raw data itself. For example, *Enterobacter* sp. strain ZOR0014 is strongly suppressed by *Aeromonas* sp. strain ZOR0001 if the two are inoculated together (Fig. 2B). Comparing *Enterobacter* sp. strain ZOR0014 abundance in fish colonized by all species except *Aeromonas* sp. strain ZOR0001 with its abundance in fish colonized by all species, in contrast, shows little difference, indicating that the *Enterobacter* sp. strain ZOR0014-*Aeromonas* sp. strain ZOR0001 interaction is strongly attenuated by the presence of the other bacterial groups (Fig. 3A).

Two additional observations also imply the presence of strong higher-order interactions in our intestinal ecosystem. Considering fish colonized by all five bacterial species, the mean abundance of each species is at least as high as would be predicted solely from direct interactions (Fig. 4D). Moreover, the diversity of bacterial species is higher than would be predicted, as all of the species occur in more than 50% of fish, contrary to prediction (Fig. 4E). Considering fish colonized by all five bacterial species, the abundance of each species is at least as high and the diversity of bacterial species is higher than the values that would be predicted solely from direct interactions.

Our finding of higher species diversity than expected from pairwise interactions in a system of several gut bacterial species is consistent with recent theoretical studies that suggest, for a variety of reasons, that higher-order interactions are likely to stabilize communities and promote coexistence. The topic of multispecies coexistence has a long history in ecology. Especially since classic work by Robert May showing that a system comprising pairwise interacting constituents will, in general, be less stable as the number of species increases (73), explaining how complex communities can exist has been a theoretical challenge. There are many resolutions to this paradox, such as spatial heterogeneity, interactions across trophic levels, and temporal variation. However, even without such additional structure, incorporating higher-order terms into general random competitive interaction models leads to widespread coexistence (45–47). Such large-scale coexistence can also emerge naturally from contemporary resource competition models (48, 74), in which cross-feeding or metabolic tradeoffs necessarily involve multiple interacting species. Intriguingly, the abundance distributions of all five of our gut bacterial species, when inoculated together, are similar to one another. The average Shannon entropy of the five-species community (H = 1.16 ± 0.24) (see Text S1 in the supplemental material) also resembles that of a purely neutrally assembled community (H = 1.61), reminiscent of dynamics mimicking neutral assembly that emerge from multispecies dynamics driven by resource use constraints (48, 75).

Our findings imply that measurements of two-species interactions among microbial residents of the vertebrate gut are insufficient for predictions of community dynamics and composition. Moreover, they imply that inference from microbiome data of interspecies interactions, for example, by fitting Lotka-Volterra-type models with pairwise interaction terms (26, 62, 63, 76), should not be thought of as representing fundamental pairwise interactions that would be manifested if, for example, the constituent species were isolated but rather as effective interactions in a complex milieu.

Our measurements do not shed light on what mechanisms give rise to higher-order interactions in our system. Likely candidates include metabolic interactions among the species, interactions mediated by host activities, such as immune responses, and modulation of spatial structure by coexisting species. Immune responses are sensitive to specific bacterial species (77) and to bacterial behaviors (78). Regarding spatial structure in particular, *in vivo* imaging of these bacterial species in monoassociation has shown robust aggregation behaviors that correlate with location in the gut (54). Given the physical constraints of the intestinal environment, we think that the modification of spatial organization due to the presence of species with overlapping distributions is a likely mechanism for higher-order interactions. Notably, both immune responses and spatial structure are amenable to live imaging in larval zebrafish (39, 40, 54). Although the parameter space of transgenic hosts, fluorescent labels, and interaction timescales to explore in imaging studies is potentially very large, such future studies are likely to yield valuable insights into the mechanisms orchestrating the strong interactions observed here. Furthermore, examination of the roles of priority effects and other aspects of initial colonization, as well as the stability of diverse communities with respect to invasion, may reveal routes for intentionally manipulating the vertebrate microbiome to engineer desired traits.

## MATERIALS AND METHODS

### Animal care.

All experiments with zebrafish were done in accordance with protocols approved by the University of Oregon Institutional Animal Care and Use Committee and by following standard protocols (79).

### Gnotobiology.

Wild-type (ABC×TU strain) larval zebrafish (Danio rerio) were derived germfree as described in reference 36. In brief, embryos were washed at approximately 7 h postfertilization with antibiotic, bleach, and iodine solutions and then moved to tissue culture flasks containing 15 ml sterile embryo medium solution with approximately 1 ml of sterile solution per larva. The flasks were then stored in a temperature-controlled room maintained at 28°C.

### Bacterial strains and culture.

The five bacterial strains used in this study, namely, *Aeromonas* sp. strain ZOR0001, Pseudomonas mendocina (ZWU0006), Acinetobacter calcoaceticus (ZOR0008), *Enterobacter* sp. strain ZOR0014, and *Plesiomonas* sp. strain ZOR0011 were originally isolated from the zebrafish intestine and have been fluorescently labeled to express green fluorescent protein (GFP) and dTomato, facilitating their identification in our experimental assays (80, 81). Stocks of bacteria were maintained in 25% glycerol at −80°C.

### Inoculation of tissue culture flasks.

One day prior to inoculation of the tissue culture flasks, bacteria from frozen glycerol stocks were shaken overnight in lysogeny broth (LB medium; 10 g/liter NaCl, 5 g/liter yeast extract, 12 g/liter tryptone, 1 g/liter glucose) and grown for 16 h overnight at 30°C. Samples of 1 ml of each of the overnight cultures were washed twice by centrifuging at 7,000 × *g* for 2 min, removing the supernatant, and adding 1 ml of fresh sterile embryo medium. At 5 dpf, the tissue culture flasks were inoculated with this solution at a concentration of 10^6^ CFU/ml. For each of the competition experiments involving 2, 4, and 5 bacterial species, equal concentrations were inoculated into the flasks. After inoculation, the flasks were maintained at 30°C until dissection at 7 dpf.

### Dissection and plating.

To determine the intestinal abundance of bacterial species, dissections of larval zebrafish were performed at 7 dpf. Zebrafish were euthanized by hypothermal shock. Intestines were removed by dissection, placed in 500 μl of sterile embryo medium, and homogenized with zirconium oxide beads using a bullet blender. The homogenized gut solution was diluted to 10^−1^ and 10^−2^, and 100-μl volumes of these dilutions were spread onto agar plates. For mono- and diassociated inoculations, tryptic soy agar (TSA) plates were used in which fluorescence could be used to differentiate up to two species. For inoculations of more than two species, Universal HiChrome agar (Sigma-Aldrich) plates were used, allowing for visual differentiation of each species using a colorimetric indicator. The abundances of each of the species in the zebrafish gut were determined by counting the numbers of CFU on the plates. These abundances for different experiments are provided in Data Set S1.

### *In vitro* competition experiments.

To determine the *in vitro* competition coefficients, all of the different pairwise combinations of the five species were grown in overnight cultures of LB medium as described above. On the following day, cultures were plated at 10^−7^ or 10^−6^ dilutions, depending on the ability to detect both species in a given dilution. Abundances were obtained by counting the number of CFU of each species on the plates. These values are provided in Data Set S1.

10.1128/mBio.01667-20.9 FIG S6 Predicted five-species distributions from the linear model. (A) Predicted (blue Xs) and measured (brown circles) absolute abundance distributions for five-species inoculation experiments calculated from the linear model for pairwise interactions for each of the five species. The means of the predicted and measured distributions, excluding nulls, are shown using boldface blue circles and brown square markers, respectively, with error bars indicating the standard deviations. The dotted line indicates the experimental detection limit of 25 cells. The predicted distributions are generated from sampling the interaction coefficient distributions as described in Text S1, while the experimental distributions comprise abundances from *N* = 202 fish. (B) The observed occurrence frequencies of each species in five-species experiments plotted against the predicted frequencies generated from the linear model. Download FIG S6, PDF file, 0.7 MB. Copyright © 2020 Sundarraman et al. 2020 Sundarraman et al. This content is distributed under the terms of the Creative Commons Attribution 4.0 International license.
