# Supplementary figures and images for: Higher-Order Interactions Dampen Pairwise Competition in the Zebrafish Gut Microbiome
Source: mBio. 2020 Oct 13;11(5):e01667-20. doi: 10.1128/mBio.01667-20 (PMC7554667; doi:10.1128/mBio.01667-20)

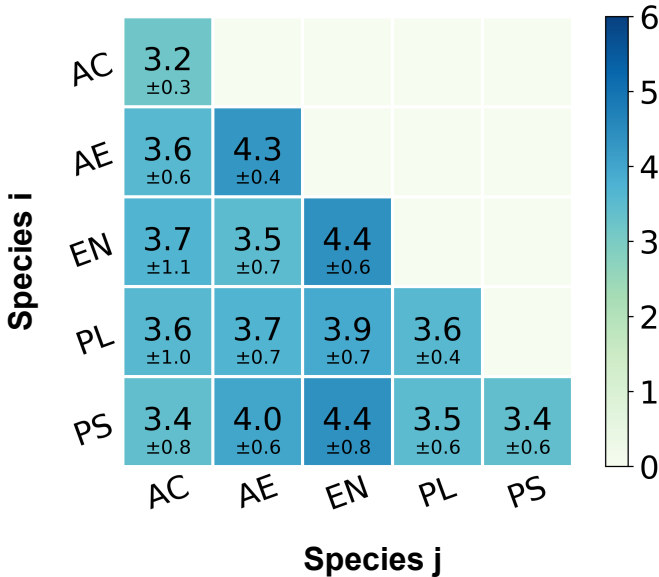

Supplement: FIG S1 [file mBio.01667-20-sf001.pdf]

**A****Species i**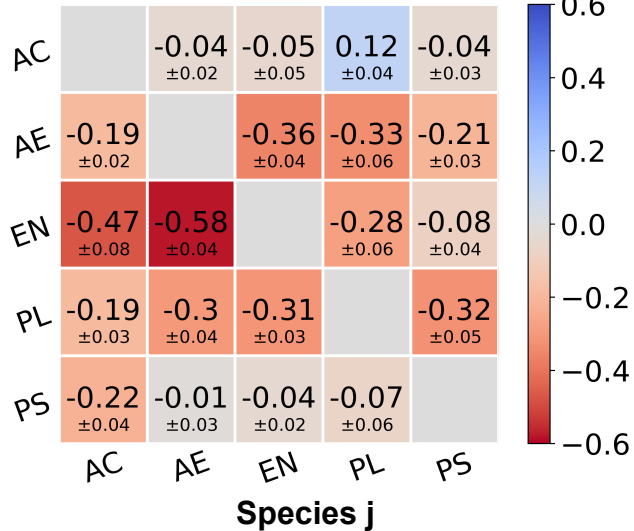**B****Species i**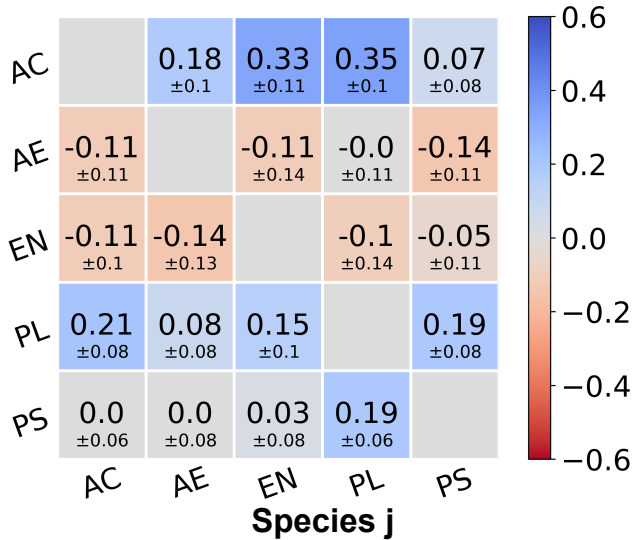

Supplement: FIG S2 [file mBio.01667-20-sf002.pdf]

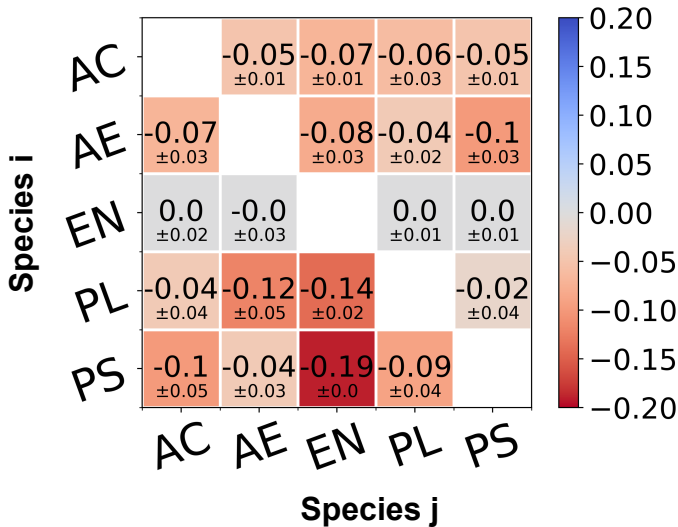

Supplement: FIG S3 [file mBio.01667-20-sf003.pdf]

$\alpha = 0.1$

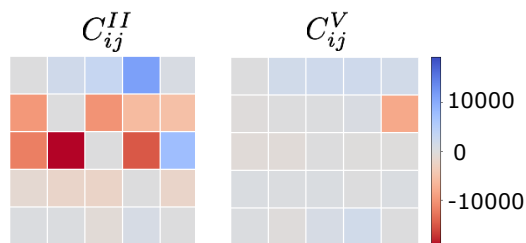

$\alpha = 1.5$

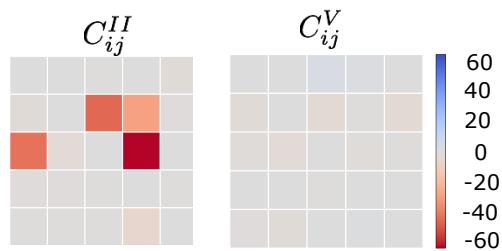

$\alpha = 0.5$

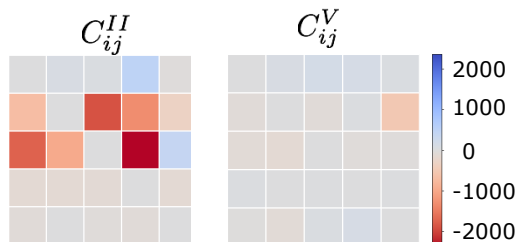

$\alpha = 2$

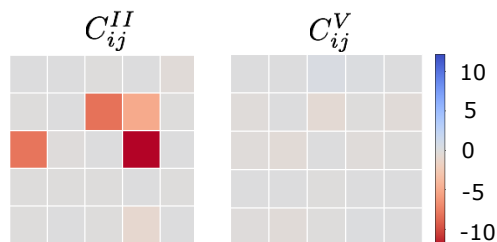

$\alpha = 1$

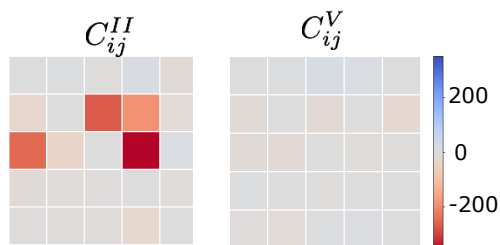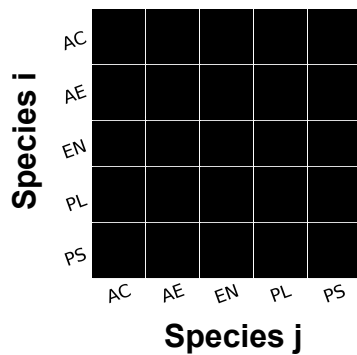

Supplement: FIG S4 [file mBio.01667-20-sf004.pdf]

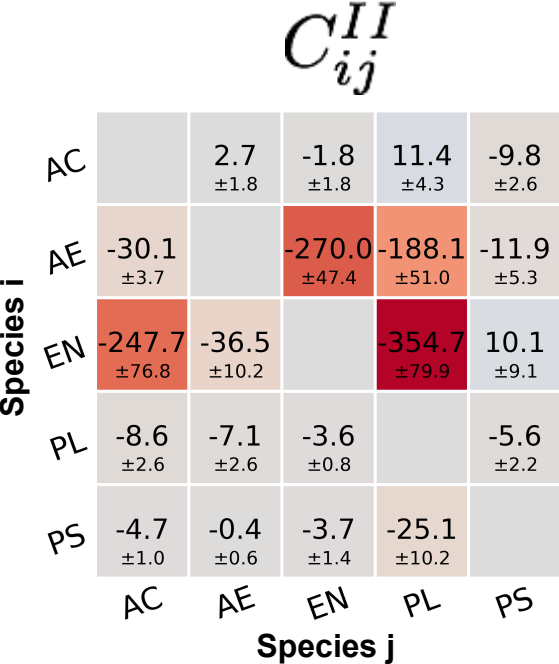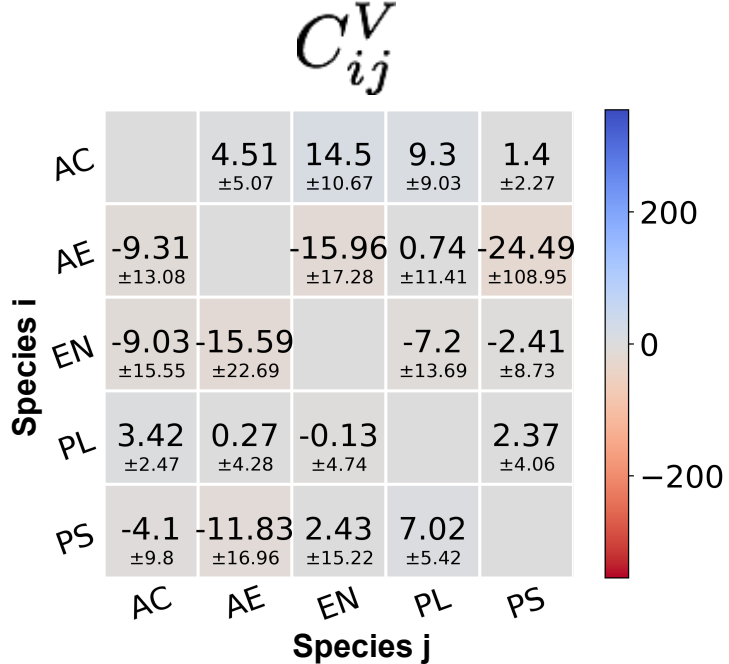

Supplement: FIG S5 [file mBio.01667-20-sf005.pdf]

**A**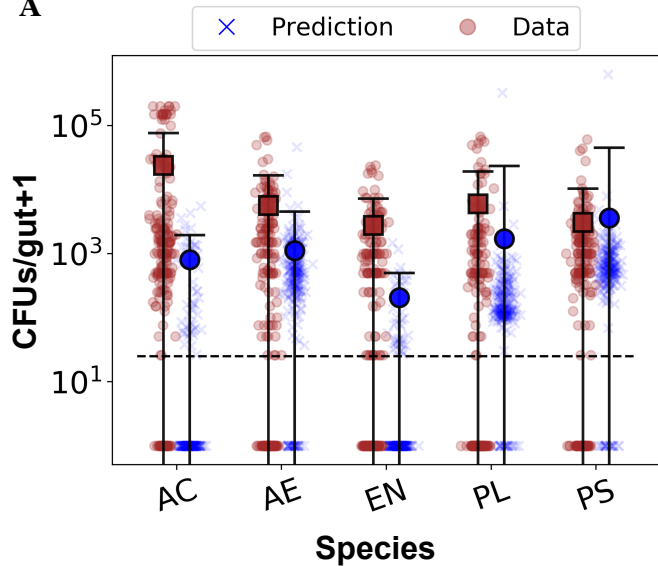**B**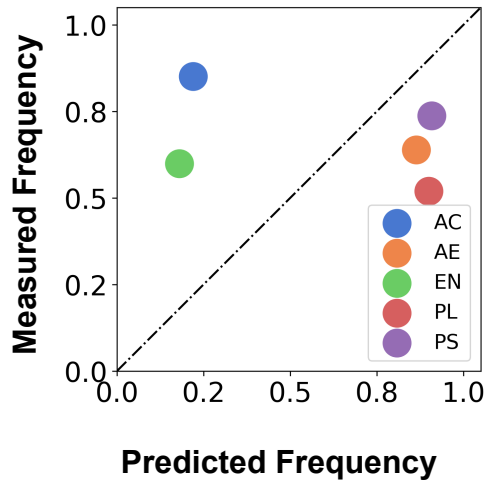

Supplement: FIG S6 [file mBio.01667-20-sf006.pdf]
