# Supplementary material for: Higher-Order Interactions Dampen Pairwise Competition in the Zebrafish Gut Microbiome
Source: mBio. 2020 Oct 13;11(5):e01667-20. doi: 10.1128/mBio.01667-20 (PMC7554667; doi:10.1128/mBio.01667-20)
Supplement: TEXT S1 [file mBio.01667-20-s0001.pdf]

Math Supplement

1 Interaction Models

1.1 Pairwise Interactions

As noted in the main text, we aim to characterize the measured abundances of microbial species with numerical parameters that characterize their interactions. These parameters may simply be phenomenological, but may also relate to dynamical population models.

Considering two species, labeled by subscripts i and j, the commonly used competitive Lotka-Volterra model describes the behavior of the populations P by the following relationship:

$$\frac{\mathrm{d}P_i}{\mathrm{d}t} = r_i P_i \left(1 - \frac{P_i - C_{ij} P_j}{K_i}\right) \tag{1}$$

where r_i is the intrinsic growth rate, K_i is the carrying capacity, and C_{ij}, defined for is the interaction coefficient, negative for competitive interactions and positive for beneficial interactions.

As steady state, $\frac{\mathrm{d}P_i}{\mathrm{d}t} = 0$, hence,

$$C_{ij} = \frac{P_i - K_i}{P_j}. \tag{2}$$

Note that this holds even if the initial factor in the Lotka-Volterra equation, $r_i P_i$ is replaced by any generic function of P_i . As discussed in the main text, each of the bacterial species examined is known to reach its carrying capacity approximately 10-12 hours after inoculation [1], a timescale considerably shorter than the 48 hours post-inoculation of our abundance measurements. It is therefore reasonable to make use of the steady-state approximation.

In our experiments, we measure intestinal abundances in di-associations, P^{II}, and mono-associations, P^{I}. The carrying capacity, K_i, is unknown. We assume K_i can be well approximated by the average P_i^I across fish, i.e. that the average abundance of a species in the absence of other species is similar to its carrying capacity in the gut. Therefore:

$$C_{ij} \approx \frac{P_i^{II} - \langle P_i^I \rangle}{P_j^{II}} \tag{3}$$

where the angle brackets indicate the mean across fish. Eq. 3 provides a simple characterization of inter-species interactions in the context of the standard competitive Lotka-Volterra equations.

As discussed in the main text, there is no reason the intestinal microbial populations must be governed by Lotka-Volterra dynamics or any linear interaction model. We therefore consider a model of logarithmic interactions and a more general family of power-law models. The logarithmic model, influenced by the order-of-magnitude changes in species abundance induced by competition, can follow from a phenomenological recasting of the competitive Lotka-Volterra model in terms of log-transformed populations:

$$\frac{\mathrm{d}\log_{10} P_i}{\mathrm{d}t} = f(P_i)\left(1 - \frac{\log_{10} P_i - C_{ij}\log_{10} P_j}{\log_{10} K_i}\right), \tag{4}$$

where we note that the first factor on the right hand side can be any generic function of P_i. Again assuming steady state dynamics and estimating $\log_{10} K_i$ by the average of $\log_{10} P_i$, the interaction coefficients are given by:

$$C_{ij} \approx \frac{\log_{10} P_i^{II} - \langle \log_{10} P_i^{I} \rangle}{\log_{10} P_j^{II}}. \tag{5}$$

which is Eq. 1 of the main text. We note that regardless of the validity of the dynamical model, Eq. 4 can be considered as a phenomenological characterization in which interaction effects are logarithmically additive (i.e. multiplicative) in population size.

Finally, we consider interactions with a power-law dependence on the size of the influencing species:

$$\frac{\mathrm{d}P_i}{\mathrm{d}t} = f(P_i)\left(1 - \frac{P_i - C_{ij}(P_j)^{\alpha}}{K_i}\right), \tag{6}$$

from which

$$C_{ij} \approx \frac{P_i^{II} - \langle P_i^{I} \rangle}{(P_j^{II})^{\alpha}} \tag{7}$$

As noted in the main text, we consider sub-linear ($\alpha < 1$) and super-linear ($\alpha > 1$) interactions. Linear interactions ($\alpha = 1$) are equivalent to the standard Lotka-Volterra model.

1.2 Pairwise Interactions in the Presence of Additional Species

To compare interactions between communities of two species and communities of more species, namely four or five, we extended the above parameterization. The standard Lotka-Volterra dynamical model for an arbitrary number of species is given by

$$\frac{\mathrm{d}P_i}{\mathrm{d}t} = r_i P_i \left(1 - \frac{P_i - \sum\limits_{j \neq i} C_{ij} P_j}{K_i}\right) \tag{8}$$

where the sum runs over species. Again considering steady-state,

$$P_i = K_i + \sum_{j \neq i} C_{ij} P_j \tag{9}$$

As discussed in the main text, we infer C_{ij}^V by comparing the abundance of species i in fish co-inoculated by a set of species excluding and including species j. Again denoting the number of species by a superscript, subtracting P_i^{IV} from P_i^V gives

$$P_i^V - P_i^{IV} = C_{ij}^V P_j^V, \tag{10}$$

from which

$$C_{ij}^V = \frac{P_i^V - P_i^{IV}}{P_j^V}. \tag{11}$$

Similar expressions follow for the multi-species interaction coefficient in the log-transformed model:

$$C_{ij}^V = \frac{\log_{10} P_i^V - \log_{10} P_i^{IV}}{\log_{10} P_j^V}. \tag{12}$$

and the power-law model:

$$C_{ij}^V = \frac{P_i^V - P_i^{IV}}{(P_j^V)^\alpha}. \tag{13}$$

The interaction matrices for calculated for the two-species experiments (C_{ij}^{II}), using Eq. 5 and four-to-five-species experiments (C_{ij}^V), using Eq. 12 are shown in Fig S2. As noted in the main text, we found similar patterns distinguishing C_{ij}^{II} and C_{ij}^V, namely weaker and more positive interactions in the latter case, throughout the full range of α examined (Figure 3D, Fig S4). The mean C_{ij} values for different values of α are provided in File S2.

2 Sampling and Parameter Estimation

For each di-association, we measured the species abundances P_i^{II} and P_j^{II} in N=10 to 30 fish and calculated C_{ij}^{II} for each according to Eq. 3, 5, 7 above. To ensure that the measured abundance changes can be attributed to the presence of the second species we ignored data for which either P_i^{II} or P_j^{II} was zero. Each set of abundance measurements provides a C_{ij} value via the above equations. To determine the most likely C_{ij} from this set, as well as the uncertainty in our estimation of C_{ij}, we make use of sampling. With 3000 repetitions, we randomly sampled 75% of each dataset without replacement and calculated the mean and standard deviation of the C_{ij}.

To determine the number of repetitions needed to accurately estimate C_{ij}, we performed the same analysis on simulated population data, drawing P_i^{II} from a log-normal distribution, using a fixed $\langle \log_{10} P_i^I \rangle$, and drawing C_{ij} from a normal

distribution of known mean and standard deviation. Using these we compute $\log_{10} P_j^{II}$ using Eq. 5, and from the simulated data we calculated the mean and standard deviation of the interaction coefficient as above. We found that sampling 75% of the dataset, ~ 2000 repetitions yield the standard deviation of C_{ij} to an accuracy of 90%. The resulting mean and standard deviation of C_{ij}^{II}, one for each species pair, are shown in the figures in the main text and Fig S2A.

Interaction parameters in multi-species experiments, i.e. C_{ij}^{V}, were similarly calculated from subsampling abundance data from both the four species and the five species experiments. The mean and standard deviation of the C_{ij}^{V} coefficients calculated using this model are shown in Fig S2B. As in the calculation of the di-association-derived C_{ij}^{II}, we considered only data from fish in which each of the species inoculated in the four and five species experiments was detected, ensuring that the effects from all other species are consistent between experiments.

In vitro interaction coefficients were also calculated using Eq. 5 from abundance measurements in pairwise competition experiments in Lysogeny broth (LB) medium. The parameters shown in Fig S3 reflect the mean and standard deviation of the coefficients from six replicates for each of the pairs of species.

3 Relative Abundance Correlations

As noted in the main text, the Pearson correlation coefficients between the relative abundances of species are commonly interpreted as measures of interspecies interactions. Denoting the relative abundance of species i (i.e. the population normalized to the sum of all species populations) in fish n as $(p_i)_n$, we calculate the correlation coefficient as usual:

$$r_{ij} = \frac{\sum_{n=1}^{N} \Big((p_i)_n - \langle p_i \rangle \Big) \Big((p_j)_n - \langle p_j \rangle \Big)}{\sqrt{\sum_{n=1}^{N} \Big((p_i)_n - \langle p_i \rangle \Big)^2 \sum_{n=1}^{N} \Big((p_j)_n - \langle p_j \rangle \Big)^2}} \tag{14}$$

where N is the total number of fish examined. Note that by construction, r_{ij} is symmetric.

4 Predicted Five-Species Abundance Distributions

We asked whether we can recreate the experimentally observed five-species abundance distributions using only the pairwise interaction coefficients from Section 1.1. Focusing first on the logarithmically additive model, we construct a predicted abundance value for each species by linearly combining the pairwise effects of each of the other species:

$$\log_{10} P_i^V = \langle \log_{10} P_i^I \rangle + \sum_{j \neq i} C_{ij}^{II} \log_{10} P_j^V \,. \tag{15}$$

P_i^V and P_j^V are the predicted abundances of species i and j respectively. This gives, for the five bacterial species in our system, five equations with five unknowns, namely the predicted P_i. To determine the distributions of predicted abundances for each of the five species, we randomly sampled each C_{ij}^{II} from the interaction coefficient distributions obtained in Section 1.1 . We performed this sampling 250 times to generate a comparable number of points to the experimental distribution (N=202). Both the predicted and the measured distributions are shown in Figure 4C. The predicted abundance distributions are provided in File S3. To mimic the experimental detection limit of approximately 25 cells, all predicted abundances below 25 were set to zero.

Similarly, we calculated predicted abundance distributions for using the linearly additive model in absolute abundance (i.e. competitive Lotka-Volterra), for which

$$P_i^V = \langle P_i^I \rangle + \sum_{j \neq i} C_{ij} P_j^V \,. \tag{16}$$

As above, we generated predicted distributions for each of the five species, shown in Fig S6 and the distributions are provided in File S3.

As discussed in the main text, the predicted distributions from both pairwise-additive models indicate that the five species are less likely to coexist than is observed experimentally.

5 Five-Species Shannon Entropy

We calculated the Shannon entropy, a metric commonly used to measure species richness in multi-species communities. This is defined as:

$$H = -\sum_i p_i \ln p_i \tag{17}$$

where p_i is the relative abundance of species i. For a neutral (non-interacting) community of N species, $p_i = 1/N$, on average. For five species therefore, a neutral model gives $H = \ln(5) = 1.61$. To calculate H from measured data, we used relative abundances of each bacteria species and calculated the Shannon entropy using Eq. 17. The mean Shannon entropy, averaged over all fish, is reported in the main text.

References

[1] Travis J Wiles, Matthew Jemielita, Ryan P Baker, Brandon H Schlomann, Savannah L Logan, Julia Ganz, Ellie Melancon, Judith S Eisen, Karen

Guillemin, and Raghuveer Parthasarathy. Host Gut Motility Promotes Competitive Exclusion within a Model Intestinal Microbiota. *PLOS Biology*, 14(7):e1002517, July 2016.
